# Intention to use personal health records and associated factors among healthcare providers in southwest Oromia region referral hospitals, Ethiopia: using the modified unified theory of acceptance and use technology 2 model

**DOI:** 10.3389/fdgth.2025.1368588

**Published:** 2025-02-25

**Authors:** Geleta Nenko Dube, Mulusew Andualem Asemahagn, Yared Mulu, Habtamu Alganeh Guadie, Mohammedjud Hassen Ahmed, Agmasie Damtew Walle, Getu Kassa Bitacha, Temesgen Ayenew Alameraw, Nega Abebe Meshasha

**Affiliations:** ^1^ Department of Health Informatics, College of Health Sciences, Mattu University, Mattu, Ethiopia; ^2^ School of Public Health, College of Medicine and Health Sciences, Bahir Dar University, Bahir Dar, Ethiopia; ^3^ School of Public Health, College of Medicine and Health Sciences, Dilla University, Dilla, Ethiopia; ^4^ Department of Health Informatics, College of Medicine and Health Sciences, Arba Minch University, Arba Minch, Ethiopia; ^5^ Deparment of Public Health, Institute of Health Sciences, Dambi Dollo University, Dambi Dollo, Ethiopia

**Keywords:** personal health records, intention to use, healthcare providers, unified theory of acceptance and use of technology 2, Ethiopia

## Abstract

**Introduction:**

A well-informed decision needs the collection of accurate and organized data, which is becoming more essential in the healthcare industry due to the increasing integration of various technologies. The literature has revealed that the magnitude of intention to use personal health records among healthcare providers is low. Consequently, this study aimed to assess healthcare providers’ intentions to use personal health records and its factors in Ethiopia.

**Methods:**

A facility-based cross-sectional study was conducted among 781 healthcare providers in referral hospitals in the Southwest Oromia region, Ethiopia. A simple sampling technique was used to select the study participants among healthcare providers. A pretested self-administered questionnaire was used to collect the data. The degree of correlation between exogenous and endogenous variables was described and validated using structural equation modeling using AMOS 26.

**Results:**

The proportion of intention to use personal health records was 57.6%, 95% CI (53.9–61.2). Factors positively associated with intention to use personal health records were performance expectancy (β = 0.325, *P* < 0.01), effort expectancy (β = 0.289, *P* < 0.01), social influence (β = 0.216, *P* < 0.01), and facilitating condition (β = 0.242, *P* < 0.01). Age (β = 0.269, *P* = 0.040, β = 0.326, *P* < 0.001) positively moderated the relationship between performance expectancy, facilitating conditions to intention to use personal health records.

**Conclusions:**

In general, healthcare providers’ intention to use personal health records were promising. Healthcare providers’ intentions to use personal health records were significantly influenced by performance expectancy, effort expectancy, social influence, and facilitating conditions. Hence, implementers need to give priority to enhancing the provision of a better system, the knowledge and skills of healthcare providers, and awareness creation among staff by providing continuous training.

## Introduction

Many e-health technologies have become available during the last two decades as countries have embraced e-health technology to support patient engagement and person-centered care objectives (1). A well-informed decision needs the collection of accurate and organized data, which is becoming more essential in the healthcare industry due to the increasing integration of various technologies (2, 3). The utilization of personal health records by individuals to access their health information is supported by legislation worldwide (4). Personal health records (PHRs) are described as “An electronic application that enables users to view, control, and transmit their health information and that of anyone they have the privilege to exchange it within a quiet, safe, and protected manner” (5). Healthcare providers use personal health records as a platform to exchange information between healthcare systems (6).

Although the terms patient portal, tethered PHRs, and electronic PHRs have been used simultaneously with PHRs in the literature, the more general term “personal health records” was used frequently in this paper. In the standard healthcare provider-patient relationship, the patient relies entirely on the physician to properly store patient data and use it for diagnosis and recommendations. To ensure this, doctors need to keep meticulous record-keeping systems (7). Personal health records can benefit healthcare providers in several ways, including making better decisions, scheduling appointments, improving patient-provider communication, enhancing patient engagement, and reducing the amount of information missed while communicating verbally. Notably, all the benefits of PHRs for providers depend on the PHRs being integrated with the provider's EHRs (8, 9). Functions of PHRs include viewing lab results, making prescription refill requests, and making appointments (10, 11).

Developing and implementing e-health services such as personal health records is one of the digital health strategies, according to the global strategy on digital health. Despite the considerable progress made by some countries, many countries still require institutional support for the development and consolidation of national e-health or digital health strategies such as personal health records (12). Nationally, Health Sector Transformation Plan II focused on improving the quality and efficiency of healthcare services by enhancing a patient-centered healthcare culture and increasing patient involvement via technology. Digitize and implement personal health records, one of the main strategic initiatives and information revolution agendas of Health Sector Transformation Plan Two (13). The Ethiopian Federal Ministry of Health has a health strategy development plan that explains its vision for equitable, high-quality, and timely health services. The ministry has recognized and designated e-health as a critical transformation enabler, including PHRs, to achieve this vision (14).

According to the literature, there have been a few studies done globally that show the magnitude of intention to use personal health records. For instance, study findings from Taiwan (15), Saudi Arabia (16), and studies from Ethiopia (17) revealed that the magnitude intention to use intention to use personal health records was low. The low personal health records (PHRs) utilization level has a high impact on the performance and quality of healthcare systems and results in decreased quality of care, poor decision-making, delays in healthcare services, and high medical errors (18).

The refusal of healthcare professionals (HCPs) to accept and promote the use of personal health records contributes to the gap between enthusiasm and usage of PHRs (10, 19).

A study carried out in the United States of America revealed that patients’ use of the My HealtheVet PHR was related to the experiences and views of HCPs (physicians, nurses, and pharmacists), and that many HCPs were not aware of the PHR's features, which led to the underutilization of the system (20). Sub-Saharan countries are comparatively more likely to lag behind in using these technologies due to the digital divide and different social issues, such as electrical power interruption, health professionals’ resistance, and ICT infrastructure (21, 22). Consequently, a poor health information system has been identified as a significant concern in the healthcare system (23). Even within Sub-Saharan Africa, Ethiopia's health information system is poorer than that of other developing countries (24).

Although there is little literature that has been conducted on PHRs, most of it has limitations related to sampling (nonprobability, small, low response rate), time period (outdated), and data analysis model (using simple logistic regression analysis) (16, 25–27). As much as my literature search capacity in Ethiopia, the magnitude of intention to use personal health record systems among healthcare providers is under-researched. A different study found that the intention to use personal health records is influenced by performance expectancy, effort expectancy, social influence, facilitating conditions, hedonic motivation, and habit. In addition, the intention to use personal health records is moderated by age and gender (27–30).

Therefore, this study aimed to present a modified theoretical framework constructed based on the UTAUT2 model to assess intentions to use personal health records and identify its associated factors among healthcare providers’ in Ethiopia. The findings of this study will be important to policy-makers, program owners, health professionals, and researchers to improve personal health records (PHRs) utilization for decision-making in healthcare practices.

The Ethiopian Federal Ministry of Health (EFMOH) has considered developing and formulating a national e-health strategy that realizes the standardization and implementation of national e-health systems, including personal health record systems in Ethiopia (31). One of the primary priorities of the national e-health strategy and the health sector transformation plan (HSTP) is introducing and utilizing e-health technology. While introducing a new system, it is important to assess and know the level of intention to use PHRs. However, there is not much evidence, primarily concerning the use of the UTAUT2 in Ethiopia (32). Therefore, this study aimed to determine the intention to use PHRs and identify factors among healthcare providers in southwest Oromia region referral hospitals, Ethiopia. This study will try to provide evidence on individuals’ perceptions and reasons for their intention to use PHRs. Healthcare providers, regional health bureaus, and non-governmental organizations are the study's main beneficiaries. The results of the study will be used for the planning and implementation of PHRs use in the healthcare system. It will be important for researchers as literature and further research in the field.

### Theoretical background and hypothesis

Venkatesh's research from 2003 to 2012 established the Unified Theory of Acceptance and Use of Technology (UTAUT), which offers a comprehensive framework to explain the acceptance, intention, and usage of information technology in organizations. It incorporates eight different models: the theory of planned behavior, the theory of reasoned action, the theory of technology acceptance, the motivational model, the model of PC utilization, the social cognitive theory, the motivational model, and the innovation dissemination theory. The UTAUT2 model is the most well-known of the models developed by recent studies to explain the acceptance of new technologies (28, 33, 34). The research questions can only be addressed when the pertinent theory or model is used as a theoretical foundation to explain users’ behavior towards the technology under study. This study proposes a theoretical framework based on the UTAUT2 to examine the intention to use personal health records because of their higher explanatory power. According to various studies, users’ intentions to utilize new technology are influenced by four constructs: performance expectancy (PE), social influence (SI), effort expectancy (EE), and facilitating conditions (FC). Consequently, Venkatesh et al. expanded on UTAUT by introducing three more determinants: habit (HA), price value (PV), and hedonic motivation (HM), in addition to individual variables like gender and age (33).

**Figure 1:**
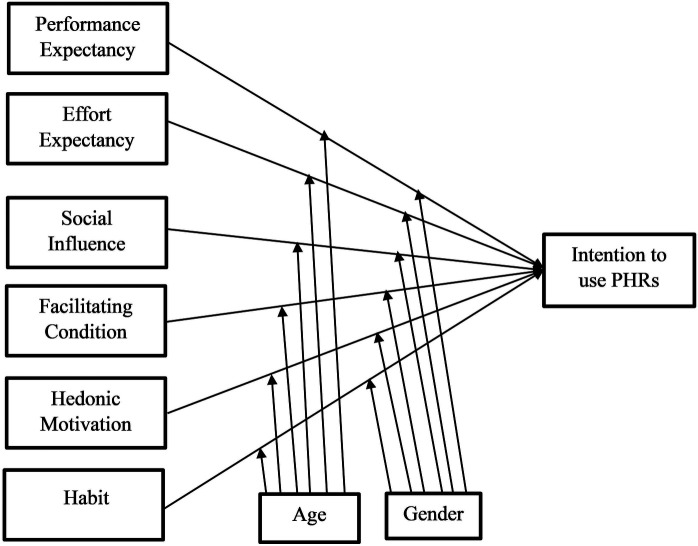
Theoretical framework for intention to use personal health records among healthcare providers in southwest Oromia region referral hospitals, Ethiopia, adapted UTAUT2 model.

Since the study's participants are healthcare providers acting as employees rather than clients, price value has been left out of the suggested model (28). For Ethiopia, we want an experimentally validated model to identify the critical implementation predictors and improve users’ behavioral intention to utilize digital health technologies, including personal health records (35). There are three sections to the theoretical research model. The first section: Performance expectancy, social influence, effort expectancy, facilitating conditions, price value, and habit are the six exogenous variables of the UTAUT2. Behavioral intention is the endogenous variable included in the second part of the model. The original UTAUT2 model used actual use as a dependent variable; however, Ethiopia has not yet adopted the anticipated technology, so this study was unable to measure actual use (33). Finally, there are the moderators, which affect both endogenous and exogenous variables, such as gender and age. The majority of healthcare providers in Ethiopia were not experienced with personal health records, so the experience was not employed as a moderator. The following is how the suggested research model is presented (Figure 1).

The ensuing subsections go into greater depth about the adapted constructs and the proposed hypotheses.

Performance expectancy (PE) is defined as “the extent to which an individual thinks that making use of the system can help them perform effectively at work” (36). A study done in Taiwan, England, and Saudi Arabia revealed a significant association between the intention to use PHRs and performance expectancy (16, 25, 27, 29, 37). A study conducted in Ethiopia showed that performance expectancy was significantly associated with the intention to use EMRs (28, 30).

H1: PE has positively impacted healthcare providers’ intention to use personal health records.

Effort expectancy (EE) is defined as “ the level of simplicity involved in using the system” (36). A study conducted in Saudi Arabia, the United States of America, Bangladesh, England and Taiwan indicated that effort expectancy was significantly associated with the intention to use PHRs (9, 29, 31, 37, 38). A study done in Ethiopia indicated that effort expectancy was significantly associated with the intention to use EMRs (28, 30).

H2: EE has positively impacted healthcare providers’ intention to use personal health records.

Social influence (SI) is defined as “the extent to which a person believes that significant others believe he or she should use the new system” (36). A study done in Saudi Arabia, United States of America, China and Bangladesh showed that social influence was significantly associated with the intention to use PHRs (25, 39, 40). According to study conducted in Ethiopia showed that social influence was significantly associated with the intention to use EMRs (28, 30). However, other studies showed that it had an insignificant effect (27, 37).

H3: SI has positively impacted healthcare providers’ intention to use personal health records.

Facilitating conditions (FC) are defined as “the degree to which a person thinks that the organizational and technological infrastructure of a system exists to facilitate the system's use” (36). A study conducted in England and Korea revealed that facilitating conditions were significantly associated with the intention to use PHRs (37, 41). A study conducted in Ethiopia showed that facilitating conditions were significantly associated with the intention to use EMRs (28). A comparative study conducted in the US, Portugal and Saudi Arabia indicated that facilitating conditions do not significantly influence the intention to use PHRs (27, 29, 42).

H4: FC has positively impacted healthcare providers’ intention to use personal health records.

Hedonic motivation (HM) is defined as “delight or exhilaration obtained while using a technology system” (33). In a study conducted in Malaysia, the healthcare providers intention to use personal health records had a positive association with hedonic motivation (43). A study conducted in Bangladesh showed that hedonic motivation was an important significant factor in the adoption of m-Health (44). However, studies from Malaysia and Indonesia revealed that the behavioral intention to use personal health records is not substantially influenced by hedonic motivation (43, 45). A study conducted in Ethiopia showed that hedonic motivation did not have a significant influence on behavioral intention to use digital health technology (28, 34).

H5: HM has positively impacted healthcare providers’ intention to use personal health records.

Habit (HA) is defined as “the extent to which people perform behaviors automatically (33).” A study conducted in Germany showed that habit was a significant factor in electronic personal health records (46). Another study done in Portugal study found that habits had a major direct impact on electronic health records portals (47). A study conducted in Bangladesh showed that hedonic motivation was a significant factor in the adoption of m-Health (44). Another study conducted in Malaysia, habit is an important factor in health information applications (43). According to a study done in the United States, habits did not significantly affect the behavioral intention to use health information applications (48). A study done in Ethiopia revealed that behavioral intention to use EMRs was not significantly influenced by habit (28).

H6: HA has positively impacted healthcare providers’ intention to use personal health records.

### Moderating effect of age

The proposed study postulated that the endogenous variable intention to use PHRs is influenced by age in connection with exogenous variables such as performance expectancy, effort expectancy, social influence, enabling situations, hedonic incentive, and habit. According to a study, younger healthcare professionals are more likely than older healthcare professionals to intend to use PHRs (36). A study done in Saudi Arabia indicated that PE, EE, SI, and FC were influenced by age on the intention to use PHRs (49). Age was not shown to be a moderating factor in a study done in the United Kingdom, since it was constant for all ages (50).

H7: The impact of PE on healthcare providers’ behavioral intention to use personal health records has been moderated by age.

H8: The impact of EE on healthcare providers’ behavioral intention to use personal health records has been moderated by age.

H9: The impact of SI on healthcare providers’ behavioral intention to use personal health records has been moderated by age.

H10: The impact of FC on healthcare providers’ behavioral intention to use personal health records has been moderated by age.

H11: The impact of HM on healthcare providers’ behavioral intention to use personal health records has been moderated by age.

H12: The impact of HA on healthcare providers’ behavioral intention to use personal.

health records have been moderated by age.

### Moderating effect of gender

As per the current study's hypothesis, the intention to use PHRs as an endogenous variable is influenced by gender in relation to exogenous variables (performance expectancy, effort expectancy, social influence, Facilitating conditions, hedonic motivation, and habit). Compared to female participants, male participants were more impacted and concerned regarding intention to use health information technology (51). A study revealed that gender acted as a moderator between social influence and the intention to use PHRs (52). According to a study done in Saudi Arabia, gender has a positive impact on performance expectancy, effort expectancy, and social influence on the behavioral intention to use PHRs (25).

H13: The impact of PE on healthcare providers’ behavioral intention to use personal health records has moderated by gender.

H14: The impact of EE on healthcare providers’ behavioral intention to use personal health records has moderated by gender.

H15: The impact of SI on healthcare providers’ behavioral intention to use personal health records has moderated by gender.

H16: The impact of FC on healthcare providers’ behavioral intention to use personal health records has moderated by gender.

H17: The impact of HM on healthcare providers’ behavioral intention to use personal health records has moderated by gender.

H18: The impact of HA on healthcare providers’ behavioral intention to use personal health records has moderated by gender.

## Material and methods

### Study design, period, and setting

A facility-based cross-sectional study design was conducted from March 25 to April 29, 2023. The study was conducted in the southwest Oromia region of Ethiopia. The southwest Oromia region is one of the known coffee-growing areas. It has three zones: Ilubabor, Buno Bedelle, and Jimma. There are three administrative districts, one special zone, 129 woredas, and 87 urban centers in the nation. Referral hospitals in the southwest Oromia region include Mattu Karl and Jimma University Referral Hospitals. Presently, these hospitals serve as referral centers for almost seven million people in the catchment area. This study was conducted at two referral hospitals in the southwest Oromia region: Mattu Karl and Jimma University Referral Hospitals, which are located 620 and 352 km, respectively, from Addis Ababa, the capital city of Ethiopia (53).

### Source and study population

#### Source population

The source populations were all healthcare providers at Mattu Karl and Jimma University Referral Hospitals.

#### Study population

The study populations were all healthcare providers working at Mattu Karl and Jimma University Referral Hospitals and available during the study period.

### Sampling size determination and sampling procedure

#### Sampling size determination

The number of free parameters in the hypothetical model determines the minimum sample size; it has been suggested that the ratio of respondents to free parameters to be estimated be set at 1:10 (54). Thus, the minimum sample required is 710, considering the 71 parameters to be estimated based on the proposed model and bringing participants to a free parameter ratio of 10. The final number of participants that is computed takes into consideration the 10% non-response rate and is therefore thought to represent the final number of participants. As a result, 781 was the final number of participants.

#### Sampling procedure

A simple random sampling technique with proportional allocation was applied. First, participants were proportionally allocated to each referral hospital. Second, participants were allotted proportionally based on their departments. A simple random sampling technique was used to select study participants from each department using OpenEpi random program version 3. Finally, 781 healthcare professionals were recruited for the study.

### Data collection tools

A structured self-administered questionnaire was employed in this study, which has been adapted from many studies on the UTAUT2 model (27, 28, 33, 34). There are two parts to the questionnaire. Part A concentrates on the socio-demographic characteristics of healthcare providers (institution, age, sex, type of profession, education level, and working experience) with six statements, and Part B centers on the 25 positive statements that symbolize the constructs included in the UTAUT2. The questionnaire comprises 31 statements in total, and a five-point Likert scale was used to measure the constructs, with 1 representing strongly disagree and 5 representing strongly agree (33). A well-structured questionnaire was prepared in the English language. The English language questions were used because there are healthcare professionals who don't know the local language in the study area. A case scenario was drafted for healthcare professionals who might not know about personal health records during the data collection period.

### Data collection procedures

Data were collected using a self-administered questionnaire. Three health informaticians who had good communication skills were recruited for data collection. Two health officers who had experience in research work supervised the data collection process. The goal of the study and how to collect data were described in a two-day training for data collectors and supervisors. To help study participants, understand the importance of the survey questions, trained investigators provided them with information about personal health records before the survey. After that, study participants were given the option to participate in the study or decline it. Any study participants who did not give their free consent were thanked for their time. Finally, the study participants completed the questionnaire after providing their free consent.

### Data quality control

Two days of training were given for data collectors and supervisors on the objective of the study, data collection procedures, data collection tools, the participants’ approach, data confidentiality, and the respondent's rights before the data collection date. The completeness of the questionnaire was checked every day by the supervisors. Data clean-up and cross-checking were performed before analysis. Before the actual data collection, pretesting of the questionnaire was conducted among 39 (5%) healthcare providers at Buno Bedelle Hospital. The pretest was conducted similarly to the actual study participant characteristics. The actual data collection was started after necessary corrections.

### Data management and analysis

The data were entered using epi data version 4.6 and exported to SPSS version 25 for further data cleaning and analysis. Using SPSS 25 software, the descriptive statistics of sociodemographic variables and the proportion of intention to use PHRs were determined, and the analysis of moment structure (AMOS) version 26 software was utilized to evaluate the model constructs through structural equation model (SEM) analysis. Mahalanobis d-squared was employed to confirm the multivariate outlier detection assumption., using multivariate kurtosis less than five, the normality of the data was checked, the range of the critical ratio was −1.96 to +1.96, and variance inflation factor less than ten, and tolerance less than 0.1 were used to evaluate multicollinearity, and the exogenous constructs had a correlation of less than 0.8. The confirmatory factor analysis (CFA) with standardized values for the test measurement model, which shows how measured variables combine to form constructs, was determined using AMOS software. As part of the confirmatory factor analysis error terms of indicators, a correlation between constructs and factor loadings for every statement was examined; thus, each statements factor loading value should be greater than 0.7 (25). The chi-square-ratio less than three, comparative-it-index greater than 0.9, goodness-of-fit-index greater than 0.9, adjusted-goodness-of-fit-index greater than 0.8, root-mean-square-error-approximation less than 0.08, and root-mean-square-of-standardized-residual less than 0.09 were employed to determine the goodness of fit of the models (25, 33, 55).

If the model misspecification exists or the model fit indices fall less than the cutoff point (0.7), when an statement falls below the cutoff point (0.7), we either delete it or use high modification indices to improve the model fit indices until the model fits with a threshold value no more than four times (56). To ascertain the degree of consistency or combination of variables in terms of the construct it seeks to measure, as well as the effectiveness with which the chosen construct statement measures the construct, construct validity and reliability were assessed. Since not every indicator has the same level of reliability, it is important to look at the composite reliability (CR) and report a value greater than 0.7. Additionally, when evaluating construct reliability, composite reliability provides higher quality results than Cronbach's alpha (57, 58). Construct reliability was determined using Cronbach's alpha, and all of the study's constructs were found to be greater than the recommended cutoff point of 0.7 (59).

The average variance extracted (AVE) method was used to determine convergent validity, with values higher than the 0.50 threshold (57). Discriminant validity was assessed using the heterotrait-monotrait (HTMT) ratio and the Fornell and Larcker criterion. When the square root of AVE for a particular construct was higher than its correlation with the other study constructs, discriminant validity was established (27, 56). The HTMT ratio value was below the required threshold of 0.9 (57).

To evaluate a structural model, 95% confidence intervals, squared multiple correlations (r2), the path coefficient, and the critical ratio were calculated to assess the relationship between exogenous and endogenous variables. A *P* value of less than 0.05 was used to establish statistical significance. Through interaction effects and multiple group analysis, the moderator, which can be a continuous or categorical variable, can change the way the exogenous and endogenous variables are related to one another (28). Age was divided into young (less than 30 years old) and old age groups (above 30 years old), respectively, because gender and age were regarded as binary variables in this study to evaluate the moderator (60). Multiple group analysis was used to test the moderating effects of factors among the hypothesized paths within the main study framework. To ascertain the moderator's impact, the chi-square difference and *p*-value between the unconstrained and structure weights were determined.

### Data quality assurance

Two days of training were given for data collectors and supervisors on the objective of the study, data collection procedures, data collection tools, the respondents’ approach, data confidentiality, and the respondent's rights before the data collection date. The completeness of the questionnaire was checked every day by the supervisors. Data clean-up and cross-checking were performed before analysis. Before the actual data collection, pretesting of the questionnaire was conducted among 39 (5%) healthcare providers at Buno Bedelle Hospital. The pretest was conducted similarly to the actual study participant characteristics. The actual data collection was started after necessary corrections.

## Results

### Socio-demographic characteristics of healthcare providers

An aggregate of 736 (94.2% response rate) healthcare providers took part in this study. The median age of the study participants was 32[interquartile range (IQR): 28–36] years. The majority (52%, 383/736) of the study participants were between the ages of 30–39 years. Approximately 62.9% (463/736) of the study participants were male, and 318 (43.2%) were nurses, followed by 132 (18%) who were physicians (Table 1).

**Table 1:** Characteristics of study participants at referral hospitals in the southwest Oromia region, Ethiopia, 2023.

Sociodemographic characteristics	Category	Frequency(*N*)	Percentage (%)
Intuitions	MKRH	184	25%
	JUSH	552	75%
Gender	Female	273	37.1
	Male	463	62.9
Age (year)	20–29	252	34.2
	30–39	383	52
	40–49	60	8.2
	>50	41	5.6
Type of profession	Physician	132	18
	Midwifery	64	8.6
	Pharmacist	50	6.7
	Laboratory	41	5.5
	Nurse	318	43.2
	Others^a^	131	18
Educational level	Diploma	48	6.5
	Degree	489	66.4
	Masters and Above	199	27.1
Working experience in years	<5	405	55.2
	5–10	224	30.4
	>10	107	14.4

^a^ Others include health officers, psychiatrists, radiologists, anesthesiologists, optometry, and physiotherapy.

### Intention to use personal health records

In this study, about 424 (57.6%) [95.0%: CI: 53.9–61.2] study participants were intended to personal health records. Regarding the intention to use personal health records, the median score was 11 (interquartile range: 9–12), the lowest score was 3, and the highest score was 15.

### Measurement model assessment

**Figure 2:**
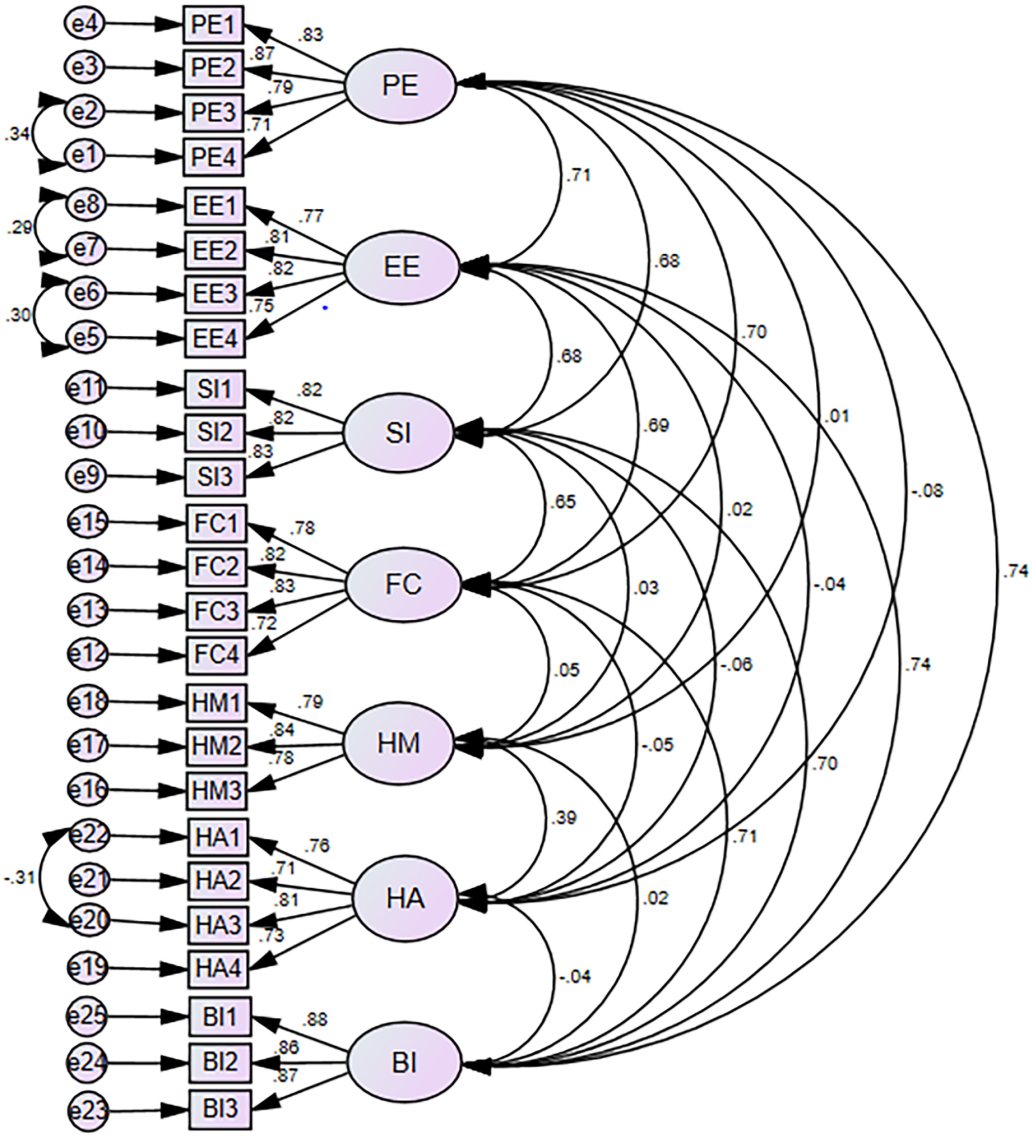
Confirmatory factor analysis of intention to use personal health records among healthcare providers in south–west Oromia region referral hospitals, Ethiopia 2023.

CFA is used to evaluate the measurement model by assessing model fit, internal consistency, convergent validity, and discriminant validity. Error term defined as an errors that arise in the predictions of an endogenous variable by an exogenous (58). To enhance model fit, we used covariate error terms with high modification indices. So, using their respective maximum modification indices, we covariate e1 with e2, e5 with e6, e7 with e8, and e20 with e22 (Figure 2). One of the main assumptions of SEM is linearity. We performed the curve estimation for all relationships in the model and determined that all relationships were sufficiently linear to be tested using covariance-based SEM.

In this study, the multivariate critical ratio did not fall between −1.69 and +1.69 (CR = 109.984), and the multivariate kurtosis value was more than five (kurtosis = 297.911). In this situation, a resampling approach that presumes a normal distribution was employed to help non-normal data through the non-parametric test of bootstrapping methods, and it determines the standard errors, path coefficients, and confidence interval significance. Five thousand bootstrap samples with a 95% bias-corrected confidence interval were used in AMOS.

### Reliability and validity of the construct

The square root Average Variance Extracted values in bold (diagonal values) are higher than other values in its column, and row, and the HTMT ratio is less than 0.9. As a result, the model's constructs’ discriminant validity had been achieved (Table 2).

**Table 2:** Discriminant validity between constructs using the fornell larcker criterion for intention to use personal health records among healthcare providers in southwest Oromia region referral hospitals, Ethiopia, 2023.

Construct	MSV	PE	EE	SI	FC	HM	HA	BI
PE	0.550	**0** **.** **803**						
EE	0.543	0.710	**0**.**791**					
SI	0.495	0.682	0.682	**0**.**824**				
FC	0.507	0.704	0.693	0.647	**0**.**787**			
HM	0.154	0.012	0.017	0.028	0.055	**0**.**802**		
HA	0.154	−0.081	−0.039	−0.056	−0.046	0.392	**0**.**755**	
BI	0.550	0.742	0.737	0.704	0.712	0.019	−0.041	**0.871**

MSV, Maximum shared variance.

The bold value provides that the maximum shared variance (MSV).

Factor loadings for each statement were investigated and the value of factor loading for each item was found to be more than 0.70. The reliability test was performed by composite reliability has values above 0.70 for all the constructs. The Average Variance Extracted values for all constructs were found to be above 0.50, which was accepted to test convergent validity (Table 3).

**Table 3:** Convergent validity between constructs for intention to use personal health records among healthcare providers in southwest Oromia region referral hospitals, Ethiopia, 2023.

Construct	Indicators	Factor loading	No of items	CR	Cronbach alpha	AVE
Performance Expectancy	PE1	0.83	4	0.88	0.88	0.64
	PE2	0.87				
	PE3	0.79				
	PE4	0.71				
Effort Expectancy	EE1	0.77	4	0.87	0.88	0.62
	EE2	0.81				
	EE3	0.82				
	EE4	0.75				
Social Influence	SI1	0.82	3	0.86	0.86	0.68
	SI2	0.82				
	SI3	0.83				
Facilitating Conditions	FC1	0.78	4	0.87	0.87	0.62
	FC2	0.82				
	FC3	0.83				
	FC4	0.72				
Hedonic Motivation	HM1	0.79	3	0.84	0.84	0.64
	HM2	0.84				
	HM3	0.78				
Habit	HA1	0.76	4	0.84	0.83	0.57
	HA2	0.71				
	HA3	0.81				
	HA4	0.73				
Behavioral Intention	BI1	0.88	3	0.90	0.90	0.76
	BI2	0.86				
	BI3	0.87				

CR, composite reliability; AVE, average variance extracted.

### Kaiser-Meyer-Olkin test

Before performing factor analysis, it is better to perform the Kaiser-Meyer-Olkin (KMO) test. The Kaiser– Meyer–Olkin (KMO) test is a statistical measure to determine how suited data is for factor analysis. The KMO test measures sampling adequacy for overall variable. According to the rule of thumb, Kaiser Meyer Olkin values between 0.8 and 1 indicate that the sampling is adequate. Hence, the Kaiser Meyer Olkin value of our study was 0.90, this indicates that the research sample was sufficient to carry out factor analysis.

### Goodness of fit

The confirmatory factor analysis results demonstrate that the fitness model’s values fulfilled the level that was required, with their corresponding values being chi-square (x^2^/df = 2.915), goodness-of-fit-index (GFI = 0.92), adjusted goodness-of-fit-index (AGFI = 0.90), comparative fit index (CFI = 0.96), root mean square error of approximation (RMSEA = 0.051), and standardized root mean squared residual (SRMR = 0.028), and *P* close (*P* = 0.338) (Table 4).

**Table 4:** Model fit indices between constructs of the intention to use personal health records among healthcare providers in southwest Oromia region referral hospitals, Ethiopia, 2023.

Fit indices	Recommended value	Authors	Model value	Conclusion
Chi-square/degree of freedom	<3	Hair et al. (2009) & Bentler (1990)	2.915	Supported
Goodness-of-fit-index (GFI)	>0.9	Chau (1997)	0.92	Supported
Adjusted goodness-of- fit-index (AGFI)	>0.8	Hair et al. (2009)	0.90	Supported
Comparative fit index (CFI)	>0.9	Bentler (1990)	0.96	Supported
Root means square error of approximation (RMSEA)	<0.08	Byrne (2001)	0.051	Supported
Standardized root mean squared residual (SRMR)	<0.09	Hair et al. (2009)	0.028	Supported
*P* close	>0.05	Bentler (1999)	0.338	Supported

### Structural equation model assessment

SEM analysis was utilized to evaluate the hypotheses after verifying that there were no significant associations between exogenous constructs and evaluating the validity of the measurement model. Collinearity has been evaluated. Collinearity can affect interpretation; it can be evaluated using the variance inflation factor (VIF) and tolerance, which show the probability of multicollinearity when they are greater than 10 and less than 0.1, respectively. This demonstrates that multicollinearity did not exist in this study (Table 5).

**Table 5:** Multicollinearity test between constructs for intention to use personal health records among healthcare providers in southwest Oromia region referral hospitals, Ethiopia, 2023.

Exogenous construct	Tolerance	Variance inflation factor
Performance expectancy (PE)	0.282	3.552
Effort expectancy (EE)	0.277	3.610
Social influence (SI)	0.340	2.932
Facilitating condition (FC)	0.315	3.172
Hedonic motivation (HM)	0.794	1.259
Habit (HA)	0.792	1.263

### Factors associated with intention to use personal health records

**Figure 3:**
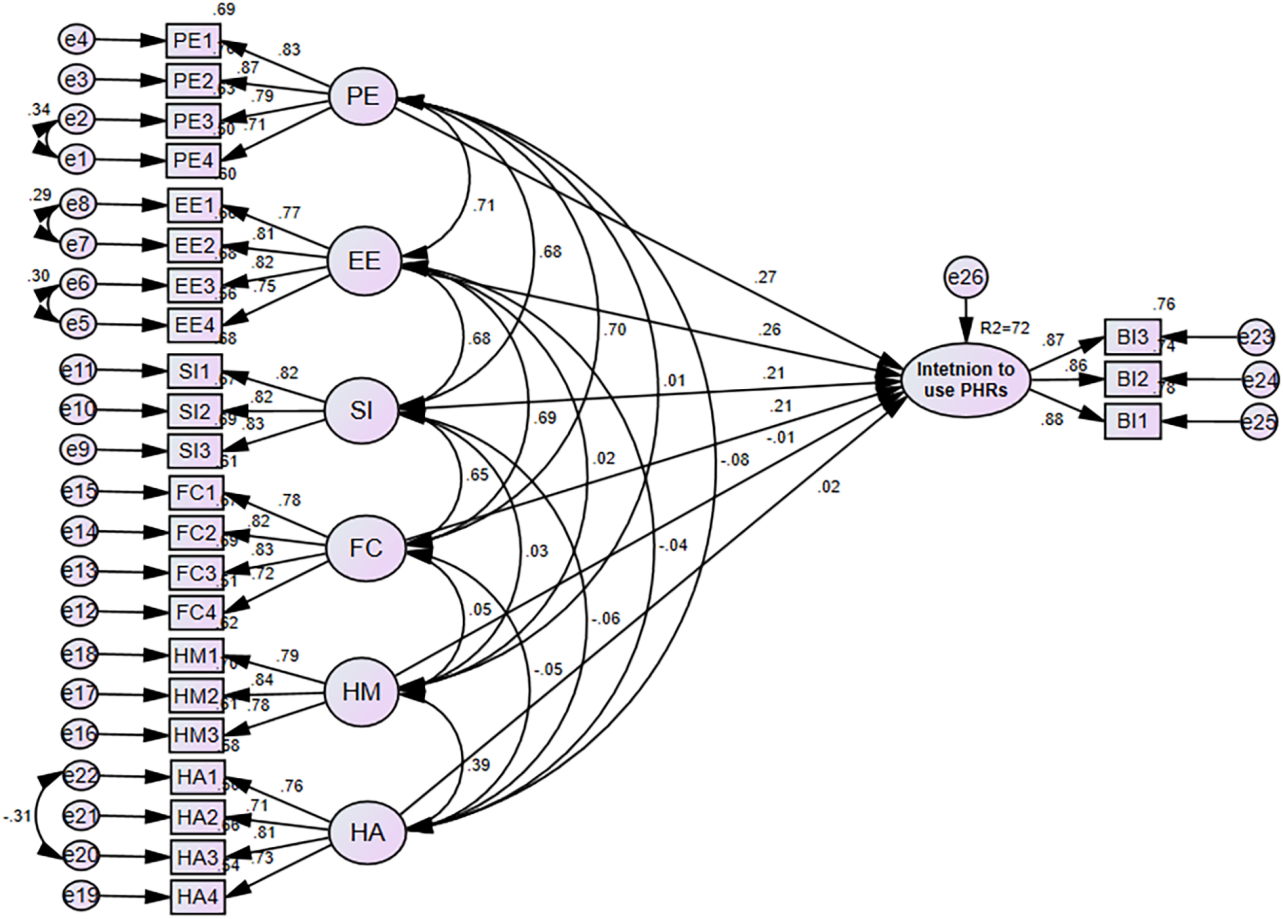
SEM analysis for factors of intention to use personal health records among healthcare providers in southwest Oromia region referral hospitals, Ethiopia, 2023.

The endogenous variable (intention to use PHRs), which has an R2 of 0.72, was 72% explained by exogenous variables such as performance expectancy, effort expectancy, social influence, enabling condition, hedonic motivation, and habit. This indicated that there was significant predictive power in the suggested model. Additionally, the findings provide substantial information on healthcare providers’ intention to use personal health records. The standardized estimate of factors for the model is displayed below (Figure 3).

According to the SEM analysis in Figure 3, Table 6, performance expectancy had a more significant impact than other factors on the healthcare provider's intention to use personal health records. The intention to use personal health records was positively impacted by having a performance expectancy [β = 0.325, 95% CI: (0.147, 0.516)], *P* < 0.01). This demonstrates that the intention to use personal health records increases when healthcare professionals’ performance expectancy rises. Additionally, the intention to use personal health records is positively impacted by effort expectancy (β = 0.289, 95% CI: [0.121, 0.449], *P* < 0.01. Likewise, this demonstrated that an increase in the effort expectancy of healthcare providers corresponds to an increase in the intention to use personal health records. In the same way, the intention to use personal health records was positively impacted by social influence [β = 0.216, 95% CI: (0.095, 0.345), *P* < 0.01], which suggests that a rise in the social influence of healthcare providers results in a rise in the intention to use personal health records.

**Table 6:** SEM analysis for factors of intention to use personal health records among healthcare providers in southwest Oromia region referral hospitals, Ethiopia, 2023.

Hypothesis	Estimate	S. E	C.R	*P* Value	95% Confidence interval		Result
					Lower	Upper	
PE→BI	0.325	0.061	5.317	0.00**	0.147	0.516	Supported
EE→BI	0.289	0.058	4.975	0.00**	0.121	0.449	Supported
SI→BI	0.216	0.047	4.559	0.00**	0.095	0.345	Supported
FC→BI	0.242	0.055	4.368	0.00**	0.068	0.415	Supported
HM→BI	−0.014	0.033	−0.428	0.668	−0.081	0.050	Not supported
HA→BI	0.019	0.034	0.565	0.572	−0.053	0.091	Not supported

C. R, critical ratio; S. E, standard error.

** *p* value is <0.01.

Finally, the intention to use personal health records was positively impacted by facilitating conditions [β = 0.242, 95% CI: (0.068, 0.415), *P* < 0.01], which indicates that healthcare providers intention to use personal health records is rising along with the facilitating conditions. In comparison, the intention to use personal health records was not influenced directly by hedonic motivation [β = −0.014, 95% CI: (−0.081, 0.050), *P* = 0.668] or habit [β = 0.019, 95% CI: (−0.053, 0.091), *P* = 0.572] (Table 6).

### Moderator effect

This study evaluated the moderating effects of healthcare providers’ age and gender on the relationship between performance expectancy, effort expectancy, social influence, facilitating condition, hedonic motivation, and habit with the intention to use personal health records. To test moderators, two model comparisons: unconstrained and structural weight models were estimated. The constrained model implies that the exogenous and endogenous variables are influenced by a moderator or significant difference in the given variable, while the unconstrained model proposes that the variable has a similar effect on influencing the relationship between the exogenous and endogenous variables. If it was determined that there was a significant difference between the two models (*p*-value less than 0.05 or chi-square difference greater than 5), Eventually, the suggested moderator variable was verified as a moderator. The findings reveal that there is no significant gender difference in the effects of performance expectancy, social influence, facilitating conditions, hedonic motivation, and habit on the intention to use personal health records. This indicates that the intention to use a personal health record system is not statistically different for females and males (Table 7).

**Table 7:** Moderating effects of gender on the intention to use personal health records among healthcare providers in southwest Oromia region referral hospitals, Ethiopia, 2023.

Hypothesis	Moderator	Path coefficient	*P* value	Model test (unconstrained & constrained model)		Result
	Gender			*Δ*X^2^	*P* value	
PE → BI	Female	0.352	***	0.222	0.638	Not
	Male	0.318	***			Supported
EE→ BI	Female	0.366	***	1.191	0.275	Not
	Male	0.282	***			Supported
SI → BI	Female	0.158	***	3.926	0.069	Not
	Male	0.274	***			Supported
FC → BI	Female	0.202	***	0.498	0.480	Not
	Male	0.256	***			Supported
HM → BI	Female	0.001	0.695	0.408	0.523	Not
	Male	−0.030	0.314			Supported
HA → BI	Female	0.034	0.354	0.064	0.800	Not
	Male	0.022	0.462			Supported

*** Significance at *P* < 0.001.

The findings showed that there were no significant differences in the effects of performance expectancy, social influence, facilitating conditions, hedonic motivation, and habit on the intention to use personal health records amongst individuals of different ages. Nevertheless, age positively moderated the relationship between performance expectancy, social influence, facilitating conditions, and intention to use personal health records. The performance expectancy was significantly greater for participants below 30 years old (young age group) (β = 0.269, *P* = 0.040) than for participants more than 30 years old (β = 0.432, *P* = 0.072). The social influence was significantly greater for participants below 30 years old (young age group) (β = 0.272, *P* < 0.001) than for participants more than 30 years old (β = 0.096, *P* = 0.110).

The facilitating condition was significantly greater for participants below 30 years old (young age group) (β = 0.362, *P* < 0.001) than for participants more than 30 years old (β = 0.090, *P* = 0.087) (Table 8).

**Table 8:** Moderating effect of age on the intention to use personal health records among healthcare providers in southwest Oromia region referral hospitals, Ethiopia, 2023.

Hypothesis	Moderator	Path coefficient	*P*-value	model test (unconstrained & constrained model)		Result
	Age			*Δ*X^2^	*P* value	
PE → BI	≤30 year	0.269	***	4.236	0.040	Supported
	>30 year	0.432	0.072			
EE→ BI	≤30 year	0.300	***	0.033	0.857	Not
	>30 year	0.313	***			Supported
SI → BI	≤30 year	0.272	***	10.337	***	Supported
	>30year	0.071	0.110			
FC → BI	≤30 year	0.090	0.087			
	>30 year	0.362	***	13.972	***	Supported
HM →BI	≤30 year	−0.028	0.467	1.306	0.253	
	>30 year	−0.025	0.326			Supported
HA →BI	≤30 year	0.057	0.120	0.556	0.456	Not
	>30 year	0.023	0.395			Supported

*** Significance at *P* < 0.001.

## Discussion

This study examines healthcare providers’ intentions to use personal health data and associated factors. According to this study, healthcare providers’ intention to use personal health records was 424 (57.6%) [95.0%: CI: 53.9–61.2]. This showed that more than half of healthcare providers intended to use personal health records for making better decisions, scheduling appointments, enhancing patient engagement, and reducing the amount of information missed while communicating verbally. In a resource-limited setting, they encounter challenges with the technology infrastructure and exhibit low socioeconomic status to adopt the new technology. This study demonstrated that healthcare providers’ intention to use personal health records was promising.

A study conducted in Ethiopia revealed scores of 39.8% and 46.5% (28, 53), which are lower than our study. One possible explanation for this discrepancy is that most study participants may not be acquainted with technologies. The small sample size (*n* = 423) and the study participants may also be contributing causes to this discrepancy.

The findings of this study were lower than those of another study done in Saudi Arabia (70%) (27). The probable causes of the discrepancies could be disparities in awareness regarding the use of personal health records. The study participants could be another likely reason for this discrepancy. Similarly, the findings of this study were lower than those of a study conducted in the Republic of Korea (72%) (61). The study participants may be the cause of this discrepancy. A potential explanation could be the focus placed on the awareness of healthcare providers about new digital health technologies that are advantageous to health. Our study's results are not as high compared to that of another study conducted in Malaysia (78%) and Canada (61%) (4, 43). The majority of study participants may not have had extensive experience with personal health records, which could account for this discrepancy. In Ethiopia, there are also concerns with low levels of developing digital health technology, inadequate e-health literacy, challenges with understanding how to use personal health records and ignorance of the significance of personal health records for healthcare services (13).

The proposed model illustrates 72% of the variance (R2 = 0.72) in the intention of healthcare providers to use personal health records. The intention to use personal health records was found to be significantly correlated with performance expectancy, effort expectancy, social influence, and facilitating conditions, showing that the intention to use PHRs was directly correlated with four out of the six path relationships in the suggested model. In comparison, the intention to use personal health records was not significantly impacted by hedonic motivation or habits. Therefore, H1, H2, H3, and H4 are accepted.

Based on the findings, the following perspectives are presented to improve Ethiopian healthcare professionals’ intentions to use personal health records:

This study found a direct relationship between healthcare providers’ intention to use personal health records and performance expectancy (β = 0.325, *P* < 0.01), which constituted the primary aspect of using personal health records. This means that healthcare providers consider personal health records to be very helpful and help them finish tasks more quickly. The findings of this investigation are in line with prior studies in Ethiopia (β = 0.39, *P* < 0.01, β = 0.298, *P* < 0.01) (28, 35), Jordan (β = 4.78, *P* < 0.001) (62), Taiwan (β = 0.078, *P* = 0.041) (9), Portugal (β = 0.285, *P* < 0.01) (42), Saudi Arabia (β = 0.22, *P* < 0.01, β = 0.17, *P* = 0.03) (25, 26), and England (β = 0.343, *P* < 0.01) (37). According to this study, PHR systems are directly linked to increases in healthcare providers’ performance. The reason for this might be that related technologies, like electronic health records, have acknowledged the value of e-health technology (PHRs). An additional probable explanation could be that PHRs’ ability to increase patient engagement, help healthcare providers make better decisions, and improve daily workflow influences them at work (62). Similarly, healthcare providers are not very familiar with personal health records because they are still a relatively new technology. For these kinds of users, performance expectancy typically has a greater impact on the intention to use personal health records (55).

This study demonstrated a direct relationship between healthcare providers’ intention to use personal health records and effort expectancy (β = 0.289, *P* < 0.01). This study indicated healthcare providers’ intentions to use personal health records could be improved if they could assume that using personal health records is simple, concise to use, and does not require much effort or skill. These findings are consistent with results from other countries, such as Ethiopia (β = 0.377, *P* < 0.001, β = 0.385, *P* < 0.05, β = 0.24, *P* < 0.001) (17, 28, 35), Taiwan (β = 0.07, *P* = 0.028) (9), Jordan (β = 4.86, *P* < 0.001) (62), Iran (β = 2.21, *P* < 0.01) (63), England (β = 0.16, *P* < 0.001) (37), Saudi Arabia (β = 0.33, *P* < 0.001) (25), and Canada (β = 0.45, *P* = 0.002) (64). A potential reason could be that healthcare professionals are already familiar with information technologies. Because of this, people might think using personal health records would be easy for them. Moreover, healthcare providers would be ready to shell out the efforts and time required to use this technology if it provided the requisite capabilities (65).

Therefore, while adopting personal health records, they should be easy to understand and operate by users for sustainable adoption of technologies in the future.

This result is in contrast to another study conducted in Saudi Arabia and Iran (29, 63). Evaluating the factors with a smaller sample size (*n* = 303) may be the cause of this discrepancy. This could be because they believe that by using PHRs, their tasks will become easier and that information can be managed clearly and systematically.

This study revealed a direct relationship between healthcare providers’ intention to use personal health records and social influence (β = 0.216, *P* < 0.01). This finding demonstrated that users are encouraged to use personal health records when those who matter to them or have a significant impact on their behavior. In another way, the findings imply that users’ acceptance of personal health record systems can be encouraged by those who directly impact them. The findings align with past studies carried out in various countries, such as Ethiopia (β = 0.18, *P* < 0.001) (28), Iran (β = 2.63, *P* < 0.01) (63), Thailand (β = 0.17, *P* < 0.001) (51), South Korea (β = 0.10, *P* < 0.001) (66), Portugal (β = 0.10; *p* < 0.05) (47), Republic of Korea (β = 0.493, *P* < 0.001) (61), and Saudi Arabia (β = 0.19, *P* < 0.001) (25). A potential explanation for this could be that hospital administration, patients, and medical professionals are pressuring healthcare providers to adopt a new system (67). Healthcare professionals may face external pressure to increase their desire and intention to use personal health records in their workplace.

Another finding of this study indicated a direct relationship between healthcare providers’ intention to use personal health records and facilitating conditions (β = 0.242, *P* < 0.01). The findings of this study suggest that healthcare professionals need to be encouraged to use personal health records by having access to resources, knowledge, and support. This demonstrates that improving personal health records alone will not increase the uptake of e-health technology; rather, the availability of resources and the knowledge required to operate personal health record systems must also be met. Despite this, the findings demonstrated the availability of the resources and skills needed to use the system, the degree of compatibility of the new system, and the support that would be provided in the event of technical difficulties. This finding is consistent with previous studies in Ethiopia (β = 0.23, *P* < 0.001) (28), Iran (β = 2.84, *P* < 0.01) (63), South Korea (β = 0.27, *P* < 0.001) (66), and the Republic of Korea (β = 0.221, *P* < 0.001) (61). The most likely explanation is that healthcare providers think they can assist patients by connecting them with specialists so they can quickly become familiar with new systems (68). Another reason could be that PHRs are seen as supported by the health sector transformation plan, according to healthcare providers. Thus facilitating conditions are crucial to encourage users (42).

Another reason could be that healthcare professionals believe they will have access to the resources and guidance needed to use personal health records at work (67). Healthcare professionals may think that completing training will put them in a better position to use PHRs because organizational preparedness and training are important components of facilitating conditions.

This study investigates if there is a gender difference in the factors that influence the intention to use PHRs. Based on the findings, gender did not moderate the impacts of performance expectancy, social influence, facilitating conditions, hedonic motivation, and habit with the intention to use personal health records. This finding is in line with previous studies showing that gender non-significantly moderates the effects of performance expectancy, social influence, facilitating conditions, hedonic motivation, and habit on the intention to use personal health records (29, 43, 69). A possible reason could be that there is no significant gender difference in the intention to use personal health records.

The results of this study showed that age positively moderated the relationship between healthcare providers’ intention to use personal health records and performance expectancy (β = 0.269, *P* < 0.001). This demonstrates that there is a substantial difference in performance expectancy between younger and older healthcare providers for those who plan to use personal health records. The findings proved that the relationship between performance expectancy and intention to use personal health records was more influenced by younger healthcare providers. This finding is consistent with the previous study conducted in China (β = 0.33, *P* < 0.001, β = 0.553, *P* < 0.01) (70) and Germany (β = 0.03, *P* < 0.001) (71). An explanation for this might be that older healthcare providers have had less exposure to emanating digital health technology. However, younger healthcare providers are more likely to be comfortable and cognizant of the importance of personal health records due to their potential exposure to the same kind of technology (70).

The results of this study revealed that age positively moderated the relationship between healthcare providers’ intention to use personal health records and social influence (β = 0.272, *P* < 0.001). This indicates that the expectations of younger and older age groups regarding social influence for individuals planning to use personal health records are significantly different. This finding suggested that younger healthcare providers had a stronger influence on the relationship between social influence and the intention to use personal health records. These results are in line with previous studies conducted in Saudi Arabia (25). The most probable explanation is that when individuals are important to them or have the power to affect their behavior, younger healthcare providers are urged to use personal health records.

The findings of this study indicated that age positively moderated the relationship between healthcare providers’ intention to use personal health records and facilitating conditions (β = 0.362, *P* < 0.001). This suggests that the facilitating conditions for those who want to use personal health records are significantly different for younger and older age groups. This finding demonstrated that older healthcare providers had a significant impact on the relationship between the facilitating conditions and the intention to use personal health records. This result is consistent with other studies in China (33). Another explanation could be that older healthcare providers tend to value the availability of sufficient support more than younger healthcare providers. A further likely reason could be that in comparison to younger healthcare providers, older healthcare providers have more difficulty adapting to new systems, which hinders their ability to learn new technology.

### Implications of the study

Lastly, the study offers both theoretical and practical insights derived from the results. The study's theoretical focus is on the proportions and factors that influence healthcare providers’ intentions to use patient health records. Any concerns regarding the acceptance of personal health records in settings with limited resources could be addressed by our findings. It serves as a baseline for researchers, particularly in settings with limited resources, because of the scant evidence regarding personal health records. Thus, our study provides statistical support for the importance of the UTAUT2 model in evaluating the intention of healthcare providers to use personal health records, and the results might apply to other nations. This study also advances our comprehension of the importance of the main factors that determine the intention to use personal health records for health management.

In practice, this study provides useful information for healthcare managers, facilities, and decision-makers to enhance the adoption and acceptability of personal health records among healthcare professionals. By making sure that healthcare services are completed more quickly, developers can improve the usability of personal health records in the healthcare industry, and upper management can help users take advantage of the relative advantages of e-health technology by supporting them, allocating resources, and delivering knowledge. In the end, the findings of this study may improve the way that technology is used, and policymakers and healthcare professionals may take them into account when deciding what additional resources to spend on the implementation of new health information systems.

## Conclusion

In general, healthcare providers’ intention to use personal health records were promising. Healthcare providers’ intentions to use personal health records were significantly influenced by performance expectancy, effort expectancy, social influence, and facilitating conditions. Out of the four determining factors, healthcare providers’ intention to use personal health records was more significantly predicted by performance expectancy. The moderation effect of gender is not significant. Age positively moderated the relationship between social influence, performance expectancy, facilitating conditions, and intention to use PHRs.

## Data Availability

The raw data supporting the conclusions of this article will be made available by the authors, without undue reservation.
